# Exploring Participants’ Experiences of a Web-Based Program for Bulimia and Binge Eating Disorder: Qualitative Study

**DOI:** 10.2196/17880

**Published:** 2020-09-23

**Authors:** See Heng Yim, Emma Bailey, Gemma Gordon, Nina Grant, Peter Musiat, Ulrike Schmidt

**Affiliations:** 1 King's College London London United Kingdom; 2 South London and Maudsley NHS Foundation Trust London United Kingdom

**Keywords:** eHealth, self-help, eating disorders, bulimia, binge eating disorder, internet-based intervention, qualitative research

## Abstract

**Background:**

Guided cognitive behavioral self-help is a recommended first-line treatment for eating disorders (EDs) such as bulimia nervosa (BN) or binge eating disorder (BED). Online versions of such self-help programs are increasingly being studied in randomized controlled trials (RCTs), with some evidence that they can reduce ED symptoms, although intervention dropout is variable across interventions. However, in-depth research into participants’ experiences and views on the acceptability of web-based interventions is limited.

**Objective:**

This is a qualitative process study of participants’ experiences of everyBody Plus, a web-based cognitive behavioral intervention, integrated into a large RCT to aid the interpretation of the main trial’s results. To our knowledge, this is the first such study in digital intervention for EDs research to include real-time feedback into the qualitative analysis. This study aims to build upon the emerging literature by qualitatively exploring participants’ experiences of a web-based intervention for BN and BED.

**Methods:**

Participants were those who took part in the UK arm of a larger RCT investigating the efficacy of the everyBody Plus intervention. Reflexive thematic analysis was completed on 2 sources of data from the online platform: real-time feedback quotes provided at the end of completing a module on the platform (N=104) and semistructured telephone interview transcripts (n=12).

**Results:**

Four main themes were identified. The first theme identified positive and negative user experiences, with a desire for a more customized and personalized intervention. Another theme positively reflected on how flexible and easy the intervention was to embed into daily life, compared with the silo of face-to-face therapy. The third theme identified how the intervention had a holistic impact cognitively, emotionally, interpersonally, and behaviorally. The final theme was related to how the intervention was not a one size fits all and how the perceived usefulness and relevance were often dependent on participants’ demographic and clinical characteristics.

**Conclusions:**

Overall, participants reported positive experiences with the use of the everyBody Plus web-based intervention, including flexibility of use and the potential to holistically impact people’s lives. The participants also provided valuable suggestions for how similar future web-based interventions could be improved and, in the context of EDs, how programs can be designed to be more inclusive of people by encompassing different demographic and clinical characteristics.

## Introduction

### Background

In many countries, the digitalization of health care services is a key strategic objective. For example, in England, the National Health Service (NHS) Long Term Plan emphasizes the need to make better use of data and digital technology in the NHS and to improve access to digital tools and services [1]. Recommendations to enable NHS staff to make the most of such innovative technologies are made in a recent independent report and a supplementary report on the *Digital Future of Mental Healthcare* [2]. In this context, it was argued that digital therapies could provide evidence-based stand-alone self-help or combined mental health interventions for service users.

In eating disorders (EDs), a growing number of studies have assessed the efficacy of eHealth and mobile health (mHealth) interventions [3-5], especially the use of structured cognitive behavioral online self-help interventions for individuals with bulimia nervosa (BN) or binge eating disorder (BED) [6]. There is some evidence that such interventions are able to reduce ED symptoms compared with waiting-list control, but comparisons with more traditional book-based self-help or face-to-face therapy are as yet relatively rare [7].

To understand people’s experiences of utilizing such web-based interventions more fully, qualitative data can be used to inform the design of complex interventions [8]. However, to date, few such studies are available. A systematic review and metasynthesis of self-help interventions for EDs [9] identified only four studies that used qualitative methodology to understand people’s experience of web-based interventions. Through meta-ethnography, six concepts related to users’ experiences of the programs were synthesized. Intervention-related factors included anonymity and privacy, accessibility and flexibility, and guidance. User-related factors included agency/autonomy, self-motivation, and expectation/attitude. These revealed some unique advantages of computer-based interventions, namely, the neutrality and the *machine-like* properties of the computer that shield the participants from other users and their online therapists or coaches. This is in contrast to potentially emotionally *hotter* face-to-face therapy, where patients might feel judged. There was a sense of increased fluidity as to where and when users could access the intervention, which required greater motivation. Health care professionals were seen as a guide, coach, or facilitator rather than a therapist. Some users viewed web-based interventions as a first step toward recovery, which might need to be supplemented with face-to-face therapy. An additional study on the views of people with EDs on online self-help interventions agrees with this point (Yim et al, unpublished data, 2020).

The Technology Acceptance Model is a framework to help understand users’ adoption and acceptance of information technology. This model posits that a potential user’s intention to use a technology and their actual usage behavior is based on the perceived usefulness and ease of use [10]. These factors have not been examined in depth in most clinical trials that focus on the efficacy of web-based interventions in EDs. Qualitative studies, especially if integrated into large-scale randomized controlled trials (RCTs), can enhance our understanding of any contextual factors as well as facilitators and barriers that influence an intervention’s acceptability, efficacy, and scalability [11]. However, in many RCTs that report on qualitative process data, these are published only after study outcomes are known, which has the potential for confirmation bias when interpreting the process data [12].

### Objectives

**Figure 1:**
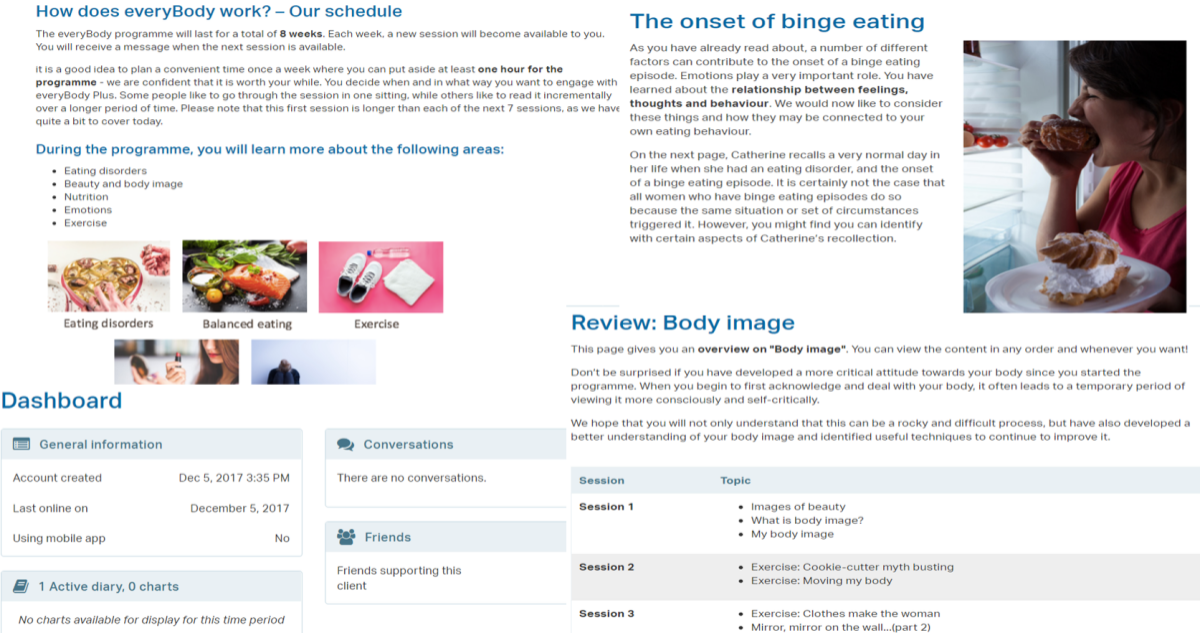
A collage of screenshots of the everyBody Plus intervention.

This study is a qualitative process evaluation of an ongoing pragmatic two-country (Germany and the United Kingdom), multisite RCT that examines the efficacy of an 8-session, guided, internet-based cognitive behavioral intervention (everyBody Plus) in adult women with BN, BED, and other specified feeding or eating disorder with binge eating (International Standard Randomised Controlled Trial Number [ISRCTN] Registry number: 12608780). Figure 1 shows a collage consisting of screenshots of the intervention. The intervention was administered to individuals who were seeking treatment or currently waiting for face-to-face outpatient treatment, aiming to bridge the waiting time. The UK arm of the intervention was developed to closely match the German program equivalent, which had been adapted from Student Bodies before the translation and editing of the English trial intervention content. Student Bodies was previously shown to be effective in reducing ED symptoms in RCTs of young women with subthreshold eating disorders [13,14]. The high usability of Student Bodies for EDs had previously been demonstrated in a mixed methods study of 9 users with usability ratings of 83.1 out of 100 [15]. In the UK trial, the main adaptations included updates on the layout of the program, replacing lengthy text passages with explanatory videos, and including written and audio testimonials of fictitious participants. Each session took approximately 1 hour to complete. Further details on the intervention and the trial can be found in the protocol [16]. The expected follow-up completion was in May 2020, followed by quantitative analysis of the RCT. Importantly, the process data are reported before completion, as recommended by Oakley et al [12].

The aims of this study are as follows:

- Explore participants’ experiences of the everyBody Plus web-based intervention
- Add to and complement the future quantitative RCT findings
- Add to the emerging literature on people’s experiences of web-based interventions for BN and BED

## Methods

The reporting of this study followed the Consolidated Criteria for Reporting Qualitative Research (COREQ) [17].

### Participants and Procedure

A total of 341 adult females were recruited into the larger RCT (ISRCTN Registry number: 12608780) from June 2016 to May 2019. Of these, 227 were from the United Kingdom. The mean age of these participants was 30.7 years (SD 10.8), and their mean self-reported BMI was 31.4 kg/m^2^ (SD 13.8). The UK participants were recruited through 15 NHS Foundation Trusts, through national and regional ED charities (Beat, South Yorkshire Eating Disorders Association, and National Centre for Eating Disorders), and through King’s College London email circulars and social media and word of mouth (for details of the RCT inclusion and exclusion criteria, please refer to the study by Vollert et al [16]). All the UK participants (N=113) allocated to the intervention condition were eligible to participate in this study in 2 different ways. First, if they had completed at least one module on the platform (n=104), their real-time feedback quotes were extracted from the platform. At the end of each module, participants were asked “Did you find this session useful?” and “We would love to hear your opinion of this session, both positive and negative comments!” where they could provide feedback. A further avenue for feedback was the group forum under the thread *Session 8: Review.* Informed consent was obtained before the commencement of their participation in the RCT. Ethical approval for the RCT was obtained from the UK Health Research Authority (reference no. 16/NW/0888).

In addition, all 113 UK participants allocated to the intervention were invited to take part in a telephone interview to discuss their experiences about the intervention via email and messaging on the online platform, where further informed consent was obtained from those who decided to participate. The analysis involved UK participants only, as the language differences hindered the ability to conduct an integrated analysis.

**Figure 2:**
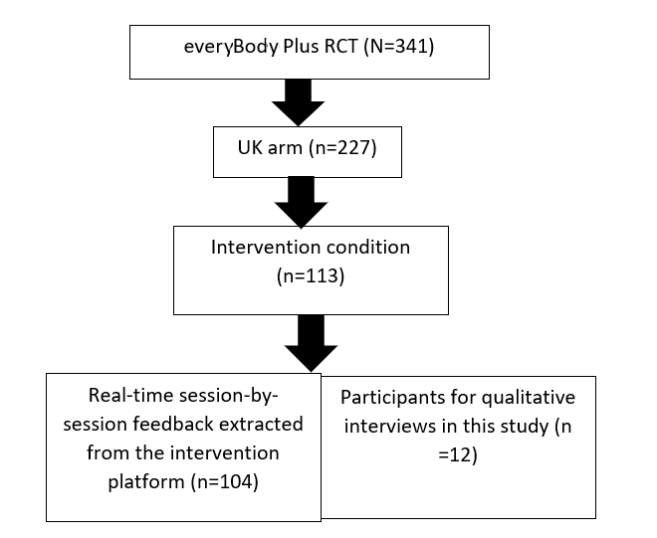
Participant and data breakdown in the current study. RCT: randomized controlled trial.

A total of 12 participants (aged between 21 and 60 years; mean 42.2, SD 13.7; mean BMI=32.7, range 19.9-50.6) consented and took part between June and August 2018. Those who did not take part did not provide any reason. Moreover, 9 interview participants completed all 8 intervention modules, 1 completed 7 modules, and the remaining 2 completed 5 and 6 modules. For clarity, Figure 2 shows the participant breakdown of this study within the larger RCT.

A semistructured interview guide was devised for the purpose of the study, which included questions on expectations, acceptability, content, and user experiences (UX; Multimedia Appendix 1). The topic guide was devised with reference to similar studies [18] and the Technology Acceptance Model [10]. Interviews were conducted over the phone by the first author SY, and the duration ranged from 20 to 60 min. No incentive was given for interview participation. Interviews were audio recorded with the participants’ permission. Field notes were made during the conversations to include the interviewer’s thoughts but were not used in the analysis.

### Data Analysis

Real-time participants’ quotes on the intervention platform and interview transcripts were transcribed verbatim by the first author SY. The two sets of data were combined and analyzed together and not linked by participants. The data were entered into NVivo software (QSR International, version 12) for analysis. Although the real-time feedback offered a naturalistic perspective of participants’ experiences of the intervention with minimal influence of the study team, the interview transcripts allowed a more in-depth, elaborate perspective for analysis.

Reflexive thematic analysis (TA) was chosen for analysis [19] because it is theoretically flexible and is useful for a relatively large set of data. The analysis broadly followed 6-step process by Braun and Clarke (2013) [20]: SY led the data extraction and transcription. First, SY familiarized herself with the data. The data were coded inductively with a mix of latent and semantic coding. Initial themes were generated after coding all transcripts, following which the codes were refined. A thematic framework was then developed and discussed with author US with illustrative examples for clarification and refinement. A critical realist approach was adopted [19], based on the idea that a real world exists but meanings are constructed and influenced by context. Although both in-session feedback and interviews rendered a relatively large sample for qualitative research, we did not aim for data saturation or complete analysis of data but instead focused on conceptual rigor [21]. We also did not include frequencies or percentages in our analysis following the guidance from Braun and Clarke [22], as the frequency of similar responses might not determine the value or relevance to the research question. Similarly, the frequency count might not be appropriate, as we could not assume the meaning of the absence of certain themes among some participants’ responses. As this approach is regarded as a *big Q* philosophically and procedurally, in contrast with *small q* versions of TA [23], multiple coders are not necessary in this approach.

### Service User Involvement

All 113 participants were invited to review the paper through the intervention platform. Two participants expressed interest in reviewing. The paper was sent via encrypted email, and the feedback was positive in general. They indicated that the paper was easy to read. The results and limitations sections were subsequently modified in response to the feedback. For example, the timing of the process study was noted as a limitation for not being able to capture how the intervention facilitated or hindered the subsequent face-to-face therapy.

### Reflexivity

The first author, SY, was aware of her multiple roles in the trial as a study coordinator, as one of the online therapists, and as an interviewer. Although this might help develop trust and closeness, as some participants had electronic contact with her during the trial, this relationship might bring bias when interviewing participants. To reduce bias, possible themes and preconceptions were discussed with the wider team. Author EB conducted interviews with the participants for whom SY was assigned as their online therapist.

## Results

### Overview of Themes

**Figure 3:**
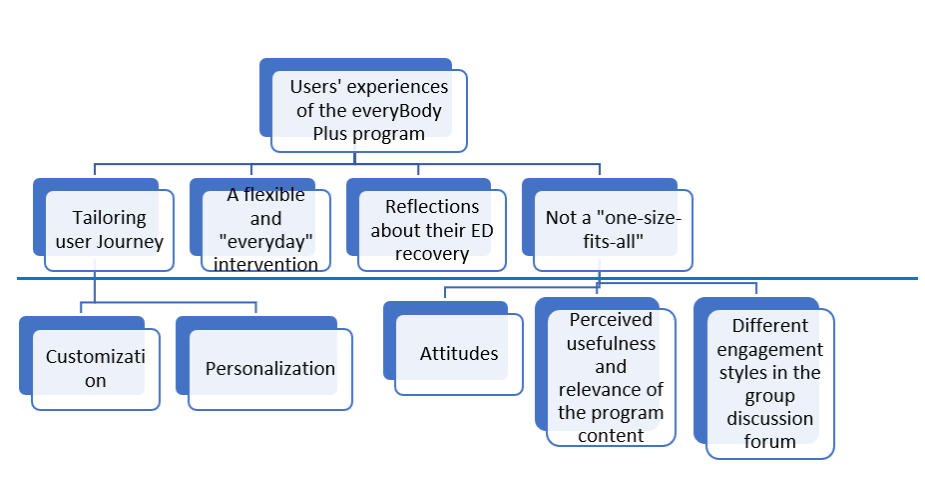
Coding tree. ED: eating disorder.

Four themes were identified, including (1) tailoring user journey, (2) a flexible and *everyday* intervention, (3) reflections about their ED recovery, and (4) *not a one size fits all* (Figure 3). We describe these themes using the illustrative samples below. Please see Multimedia Appendix 2 for a detailed and comprehensive set of quotes.

### Tailoring User Journey

This theme was closely related to the design and UX of web-based interventions in which participants expressed a strong desire for a more tailored, personalized user journey. A varied usage pattern was shown: although some participants used the program whenever they had time, on the go, or whenever they received a reminder, other participants went through the program at dedicated times.

The participants had a mixed UX. Some considered the interface easy to navigate and technical issues were minimal, whereas others pointed out the less user-friendly aspects. For example, one participant said on the group discussion forum:

I also found the UX really bad. It’s like eating a lovely biscuit that has sand in it. It doesn’t matter how lovely the biscuit is, the sand ruins it.

Group forum, GF

Sessions were seen as too lengthy (each session could take around an hour to finish), especially for those with poor concentration. For some, self-identified perfectionist tendencies meant that the tasks became “stressful to try to absorb” or even overwhelming to cope with.

Two subthemes were identified: (1) customization and (2) personalization. Customization allows users to adapt features according to their preferences. One of the features was notifications or reminders. In general, participants welcomed the email notifications reminding them, for example, to complete self-monitoring diaries or be notified when a new message was received. Nevertheless, participants seemed to prefer to be given the option to adjust the frequency (eg, daily or weekly) and the type of notifications (eg, text or email) to suit their needs:

(The) programme should fit around the user more, rather than the user having to fit around the programme.

GF

Currently, the program allows the adjustment of the frequency of the reminders and has the option to switch off the notifications. However, there was no SMS notification, and participants could not adjust the receiving time of the notifications.

Personalization was another common factor influencing the UX. Participants appreciated the use of multimedia such as text, pictures, and audio to suit different learning styles. In addition, many participants wished for a bookmark or personal route functionality, where they could immediately access the page they last visited, as the sessions were long and too cognitively demanding to finish in one go. A participant contended:

I don't like about the user-friendliness is that if I close the page partway through, even if I do an intermediate save, next time I open the session in a new window I must start from the beginning and click through all the pages to where I previously was.

In-session feedback, SF

Participants showed different preferences regarding the type of technical device used. Different motivational factors were at play when using mobile phones or laptops. Mobile phones seemed to be suited for doing the program while *on the go*:

When I am out, I don't take my laptop with me...my phone, I will just bring my phone...that I can just log in and do the main thing, like the diary, symptom tracker, get hold of people, so it does definitely help having an app.

P9, interview, I

Using the intervention on a mobile phone might also be more private, especially in households where a laptop was shared among the family members. However, an advantage of using a laptop was to allow a space to reflect, as users may easily get distracted when doing the program on the go. For example, a user said:

...going onto a laptop and doing some kind of regular, some sort of like a class or something, actually made it, gave it a bit more weight, made it feel like I was taking it a bit more seriously, so I personally found doing it via a laptop would probably be better in terms of my level of engagement.

P9, I

Furthermore, an issue highlighted by participants was that the mobile version did not include all the intervention materials:

I don't think you could access it on your mobile in the same way, it was just stripped. You have to go onto the computer to do some of it.

P7, I

### A Flexible and Everyday Intervention

The online nature of the program made it easy to embed the intervention into their everyday lives:

I found sometimes during therapy though, it became like a silo, separate from your normal life. Whereas this, because it became part of your normal life, you could think about it, think about reflecting on aspects of your life, whilst you are living it almost.

P7, I

They were able to flexibly go over the notes whenever and as many times as they wanted to (eg, “I often struggle to remember things discussed during therapy sessions”). The idea of engaging with the program on an ongoing basis through the program content and diary logging, reflecting on oneself regularly, also created a sense of continuity of self-improvement. For example, as participant 11 put it:

And I think, what I like about it, because you’re doing these diaries weekly, you are working on yourself all the time.

However, despite the advantages of the guided self-help intervention being online, additional downloadable or offline materials were still preferred by some participants. In this way, they could go through the materials at their own pace; otherwise, the process might become “too quick” and “difficult” (P5, I).

### Reflections About Their ED Recovery

Many participants described the helpfulness of the program (eg, “Lots of info. A lot to take in. Helpful reflection and realisation.”) in relation to 4 broad areas: (1) cognitive, (2) emotional, (3) interpersonal, and (4) behavioral aspects.

Cognitively, the program helped them gain new understanding and insights about their ED. One participant said:

I didn’t realise an ED was related to black and white thinking, to me that was part of my other diagnosis (borderline personality disorder), I didn’t realise it impacts my eating at all.

P9, I

Some mentioned specific program elements as helpful, for instance:

I can see that I have changed my attitude to exercise...now it is about contributing to more mental health and physical wellbeing...

P6, I

Nevertheless, some thought that the program did not offer any new information. Others thought it was still important to be reminded even though they were aware of the information:

I have seen and done some of these things before but they are equally valuable and need to be returned to again and again. I realise how obsessed I have been and still am to a degree with my body and how shame and disgust still rule me even though I have spent most my life with eating issues.

IF

Emotionally, participants mentioned being better at regulating their difficult emotions and gaining self-confidence and acceptance. One person described “feeling comfortable in her own skin now, which was an odd feeling” (P9, I). In relation to interpersonal relationships, one participant thought she had learned to deal with people’s comments about her instead of resorting to binge eating, and some mentioned being able to participate in social situations that involved food, such as family meals.

The practical techniques introduced in the program, such as the use of a reflection diary, mindfulness, and carrying out behavioral experiments, were seen as useful by some participants in affecting behavioral changes. Specific targeted behaviors included having fewer binge eating episodes as well as examining and reducing avoidance behaviors. For example, P3 said:

I ended up having like 6 weeks which I haven’t binged at all, which was a massive achievement to me.

Participants appreciated the action-oriented nature of the program:

I found that quite helpful, because you are putting it into practice. You are not just reading, you are doing something.

P1, I

### Not a One Size Fits All

Participants’ attitudes to and views of perceived usefulness and relevance of the program content were mixed. They also showed different engagement styles in the group discussion forum. As P2 noted:

People have different pasts and some have been struggling for years and years, and for some of them it’s a wake-up call...knowing that this isn’t best for all.

Participants were in different stages of recovery, and their EDs were maintained by different factors. It could be difficult for a program to be “geared around different people, different levels, identifying the balance” (P3, I).

Participants held a spectrum of attitudes toward engaging with the program as well as communicating with their online therapist. Several participants pointed out that the delay in receiving help from the NHS prompted them to try other forms of support:

Yeh, I’m one of the lucky ones, because if not I’d still be waiting, you know its 14 months, I waited for any kind of help and you know and that’s a long time without help and I can see why people with eating disorders whichever one it is, end up, you know, doing silly stuff, self-harming or committing suicide, it’s because you’ve just got no one to help.

P12, I

To some participants, the intervention felt like a “God-sent,” to “help kickstart things again” (P4, I). Some held a curious stance, wanting to try something new:

I just really wanted to do it, you know, it was something I hadn’t heard about before and just really wanted to do it.

P11

Others held a more ambivalent or skeptical view. Some participants hoped for more intensive and face-to-face help, especially those who considered themselves as “very, very, seriously unwell.” P10 also argued that as the program was intended for people who were on the waiting list for therapy, knowing that she would be receiving therapy soon, she “didn’t invest in the programme a lot,” but instead she was just “reading, ticking the box, that kind of thing.”

Divergent attitudes were also held regarding the therapist-user interaction. It was acknowledged that although this form of intervention helped reduce the power imbalance between the user and the therapist and kept users accountable and on track, the flip side was that the remoteness of the therapist-patient interaction diminished the trust in and the sense of *human-ness* of the therapist. To illustrate, some participants mentioned that they did not feel like a patient when accessing the program:

I think that power imbalance when you are in therapy, almost keeps you with that eating disorder, whereas this doesn't because I found it quite empowering, because I was in control, I could have clicked on this module or not, take the information or not, but it was different for me.

P7, I

On the other hand, other participants found the online therapist difficult to relate to as they did not know who they were. Although some expected to receive some *expert help* even though it was not *an in-depth therapy*, they were doubtful about the *therapist’s* qualifications and wondered if:

It was a very clever algorithm because the responses were very packed and formulaic...Maybe it's something about online that it's very hard to kind of set up that relationship...I admit it must as well be it's been hard for me to get past my own prejudices.

P2, I

In relation to the program content, the difference in the perceived relevance and usefulness of the program was further complicated by issues around intersectionality, such as *gender, discrimination, prejudice, and disability*. P2 felt the program did not capture the complexity of the issue, and the program needed to “acknowledge that society impacts on women in different ways, and different people experience it in different ways.” Comorbidity also influenced participants’ ability to work on the program. For example, P1 said that it is not that easy for her to take the learnings onboard when her mood was low, and she felt depressed.

In particular, participants who were older (over 50 years) and those living in larger bodies felt they were not well served by the program, as reflected in the examples, case studies, and exercises. One user commented:

I feel the course has been totally dismissive of any problems that come with obesity. It is not represented in any of the examples given, any questions asked or any exercises offered...A lot of it seems based on the idea that we should just accept our body. Well, I'm currently walking past posters from Cancer Research telling me that OBESITY is one of the largest causes of cancer, and I'm meant to ignore that in favour of feeling good about my body, am I?”

SF

The other example was shown in the case studies, where people with BED did not find that the case studies resonated with them or saw the photos used as being *glamorized* and not *authentic* but *imaginary.* Participants who were older felt that the program seemed to target a younger audience, a participant expressed that “there was no elderly women in the pictures in the program” For example, P7 expressed the following:

It seems slightly more geared towards younger women than older women, say those little videos and everything...Getting old with an eating disorder is a different challenge in itself...I think I hear things about women my age with an eating disorder, it always feels a little bit like it's been written off, like there's no hope after a certain age if you have it for a certain amount of time that it will be with you for life.

The series of mirror exposure exercises in the program exemplified the issue about the relevance of the program to some extent. Some mentioned that those exercises were clearly geared toward people with normal weight. That said, most participants found the mirror exercises challenging, although some found them useful in boosting self-confidence and self-acceptance. A user (P9, I) mentioned “at the end of the day, anything that’s worthwhile isn’t gonna be easy.”

Furthermore, some participants felt that attractiveness was overemphasized in the program. Users disagreed with the focus and felt they did not “give a damn about the media portray(al) of what an ideal body shape or weight is” (SF). Others did find the focus on external factors useful. For example, a user mentioned that it made her realize how her “distorted body image was in many ways the by-product of a distorted beauty ideal” and that recognizing that this was part of a long socialization process gave her a degree of comfort.

Participants who found the program unrelatable and not very useful shared reflections on its potential to exacerbate unhelpful or distressing thoughts and behaviors. A user posted on the platform:

I had become very depressed and had begun to think about self-harming as a way of relieving the tension and anxiety I was feeling. I realised a lot of that was coming from the exercises we had been doing as part of the programme, and my frustrations with the programme.

GF

One interviewee decided to stop doing the program. However, there were also participants who found the program comprehensive and “didn’t appear to have anything lacking” (P4, I) and found the case studies engaging and useful.

The inability to relate to the program was also shown in the group discussion forum, acting as a barrier to engagement. Some participants did not feel included or that they fitted in the forum due to having different perceptions of their issues or not wanting to upset others. A user said:

I am not a big girl, I am a normal size, I am trying to join in a conversation about how you feel about how you look when you know that there are people out there, that battling harder in a way, because they are so large. I just couldn't feel I could do it, so it's difficult when there's being such different from other people using the forum.

P4, I

A myriad of engagement styles on the group forum was displayed. Some people used the forum to support others and be supported, which helped them to feel less alone (P11, I). Other users described being a *lurker* in the group, reading others’ messages as opposed to being actively engaged. For instance, one of them mentioned that this was not for her and that she never wanted to participate in anything by writing or asking for support (P10, I).

## Discussion

### Principal Findings

In line with the stated aims of our study, namely, to understand participants’ experience of the everyBody Plus web-based intervention, to add to and complement future quantitative RCT findings, and to add to the emerging literature on people’s experience of web-based interventions for BN and BED, our TA generated 4 themes:

1. Tailoring the user journey: users had mixed feedback regarding the UX and the design of the program and expressed that the web-based intervention should be both customized and personalized.
2. A flexible and * everyday* intervention: the program’s online nature favored flexibility and continuity to embed the program into daily lives.
3. Reflections about their ED recovery: participants reported positive impacts of the program from a cognitive, emotional, interpersonal, and behavioral perspective.
4. Not a *one size fits all*: attitudes regarding the program content (case studies and the group forum included), usefulness, and relevance were divergent. In particular, participants who were older and had a larger BMI held more negative views.

Many of the comments encompassed within these themes speak to more than 1 of our 3 study aims. We discuss the findings from each of the themes in the following paragraphs.

In relation to theme 1, the UX of the program, concerns over lengthy content alongside a lack of a bookmark function to track users’ progress, reduces the perceived ease of use, as postulated in the Technology Acceptance Model. These findings highlight the need for using in-depth service user involvement in co-designing interventions before embarking on clinical RCTs. For example, Graham et al [24] argued for the importance of design research methods (eg, *think-aloud* protocol, ethnography, and user testing) in the development of web-based interventions for EDs. In this study, some of the problems identified here with UX could perhaps be prevented if a formal UX study had been carried out before the RCT; yet, due to aligning with the German version of the intervention and time constraints, a prior study was not done. Although we did not conduct a formal usability study here, our participants’ comments suggest a much more mixed usability experience than that found by Nitsch et al [15]. This may be because in the Nitsch et al [15] study, participants were women aged 18-25 years with an interest in improving body image and reducing disordered eating behaviors, who were recruited via the internet or social media and who were reimbursed for participating in the study. Thus, their views may not fully reflect those of people with clinical EDs included here. A limitation here was that the mobile version did not include all the intervention materials that were accessible on the laptop or desktop version; thus, we could not firmly draw any conclusion on the perceived relative merits of mobile or web versions. However, the analysis reflects that rather than designing an intervention as either *mobile-first* or on the web, a hybrid approach could be adopted. Participants pointed out the different nature and usage patterns of mobile phones and laptops. The findings of this study echo a previous study by Morrison et al [25]—they found that the mobile app content was typically used on-the-go and browsed for shorter periods. Different elements of the program could be augmented on mobile or laptop versions, and the choice should be promoted, which also includes having an option to download the materials or have an offline reading. Participants’ feedback about the laptop version, making them take the intervention more seriously and allow them more space for reflection, matches the findings of Dennison et al [26]. These authors reported that apps were seen as more *disposable* rather than a long-term commitment. Although convenience is an important factor for adoption in digital health intervention [15], it seems that for sustained engagement, the benefits of using a laptop need to be considered, especially when the program materials are complex and require active reflection.

In relation to theme 2, the flexibility of the web-based program transcends geographical and time restrictions, indicating an advantage of this form of intervention delivery, as previously noted in other qualitative studies (refer to the review by Yim and Schmidt [9]). A further advantage is the ease with which the intervention is integrated into people’s daily lives. As the *therapy time* is not scheduled, flexibility allows participants to access the intervention at any time, including when they are struggling with their symptoms. Completing a weekly symptom diary also gives a momentum to work on making psychological and behavioral changes. This advantage has not been mentioned in previous studies on web-based interventions for EDs. Andreassen et al [27] described a changing spatial configuration of intervention delivery from clinical spaces into domestic spaces in eHealth and postulated that this might make the difficulty more pronounced at home. In the current context, perhaps the change in therapy space is beneficial as users could reflect and access support while *living it.* This supports the concept of agency synthesized in the meta-ethnography by Yim and Schmidt [9], where users have more control over the *therapy* and hence their recovery.

Findings from theme 3, namely, participants’ reflections on their EDs are broadly consistent with meta-analysis by de Vos et al [28] regarding the criteria for ED recovery. In addition to ED symptom reduction, participants mentioned psychological, behavioral, and interpersonal changes. These reflections seem to be more comprehensive than themes identified in previous studies such as *improvements in bulimic symptoms* [29] and *changes in ED symptoms* [30], thereby increasing the perceived usefulness of the program. Indeed, the impact of EDs goes beyond cognitions and behaviors, with individuals with EDs experiencing interpersonal difficulties [31] and difficulties in emotional regulation [32]. Hence, the results of this study suggest that this web-based program has the potential to encompass a more comprehensive approach and to enable people to make holistic changes and improvements in their lives.

In relation to the fourth and last theme, similar to previous qualitative studies (refer to the review by Yim and Schmidt [9]), very heterogeneous attitudes and feedback were found. The dialectic of *anonymity* and *safety* versus *remoteness* and the *robotic* quality of web-based interventions highlights the idea that the computer can be perceived as a shield and buffer, yet could also be hard for participants to relate to. This was compounded by the complexity of the diverse engagement patterns in the group forum. Our previous study on people with EDs’ and their carers’ views on online self-help discussed the importance of interaction among participants and the community formed in the group discussion forum to break the isolation (Yim et al, unpublished data, 2020). However, this study reveals a more complex picture. We show the risk of further pushing people away due to not being able to identify with others in the group or fear of receiving unhelpful comments. Such findings are similar to qualitative studies on group cognitive behavioral therapy for EDs, whereby participants also commented on the composition of groups and how feeling different from others can interfere with how supported they feel [33]. The division was particularly felt among users of different body sizes and the extent to which they engaged in compensatory behaviors. This raises questions around how a supportive community could be formed as well as how group forums could be moderated to foster inclusivity of people with different characteristics.

### Strengths and Limitations

To our knowledge, this qualitative study is the first process study within a larger RCT that included both in-session feedback and interviews in the analysis of a digital intervention for EDs. The incorporation of real-time feedback from all participants (N=104) was a key strength, ensuring representativeness and generalizability of our findings. However, the sample size of the interview component of the study (n=12) was small, and the interviewees were not fully representative of the whole sample in some aspects, as they were older and a high proportion had completed the full intervention when compared with the 36.3% (41/113) intervention completion rate of the UK sample in the RCT. When considering its limitations, themes relating to how the program facilitated or hindered face-to-face therapy were not generated. It would be useful to understand how the program might be synergistic or in conflict with the therapists’ approaches when participants were off the waiting list, as the intervention was intended to *bridge* the waiting time between referral and face-to-face treatment. One should be cautious that the results of this study were based on data from the UK participants in the everyBody Plus trial only and may not fully represent the experiences of people from other countries using the everyBody Plus intervention in different cultural or health care contexts or those of people using other web-based interventions for EDs. Additionally, this program was designed for adult females, and the scope of feedback excludes the experiences and views of male users of web-based interventions. Nonetheless, this study raises several important implications for the RCT and for research into eHealth for EDs in general.

### Implications for Future Research and Practice

#### Implications for Digital Interventions for EDs in General

Research on intervention efficacy and effectiveness needs to consider the program layout and usability, and design research methods [24] should be adopted before any large-scale RCT to minimize technical issues affecting intervention uptake and adoption. Participants’ different preferences for mobile or laptop use suggest that instead of designing for either eHealth or mHealth, programs should be designed as a hybrid, giving users a choice. Some features that are more routine and can easily be done *on the go*, such as self-monitoring diaries, could be designed as *mobile-first*.

Clinically, this, like other web-based interventions that may be used while people are waiting for face-to-face therapy, shows potential for increasing access and preparing people for therapy through teaching cognitive behavioral principles and helping them reflect on their thinking and behaviors. Clinicians need to be aware of and monitor people’s motivations, explaining the purpose of the program as a treatment intervention in its own right, to avoid people treating this as a *tick-box* exercise knowing that they have already secured face-to-face therapy sessions.

In addition, our findings cast doubts on the possibility of designing a program that is inclusive of and speaks to the full range of people with binge eating–related issues. Another issue any web-based program needs to consider is the potential harm group forums may pose to people who do not identify with others in the groups, as found here. Future research and design of web-based programs may want to consider using more tailored group forums, depending on key clinical characteristics. For instance, having separate forums for those who do and do not use compensatory behaviors, such as self-induced vomiting, could allow participants to feel more included and make the forums more relevant to their specific needs.

#### Implications Specific to everyBody Plus and the RCT

For the everyBody Plus intervention specifically, the results reveal the need for future iterations to improve the UX. The Technology Acceptance Model identified *ease of use* as a factor for technology adoption. Improving the UX will be paramount to increase adoption and adherence. In the version used in this study, there was no way for participants to return to the page they last visited or to bookmark information. As the modules were perceived as long, the bookmarking function was important. The mobile version needs to be improved as some of the training materials are accessible only via a laptop or desktop computer.

At the time of writing this paper, the main findings from the trial in relation to the clinical outcomes are yet unknown. Although the main analyses of the RCT (from which the present data are drawn) are prespecified in the trial protocol [16], qualitative data such as those presented here may offer ideas for additional, more exploratory (moderator) analyses and offer a nuanced understanding of reasons explaining the attrition rate in the main intervention.

### Conclusions

In conclusion, this study has highlighted what participants find helpful and positive about this web-based intervention and provided suggestions for improvements to the future design of such interventions. This will help inform and complement the upcoming analysis of the RCT to allow us to better draw conclusions on the next steps for the web-based intervention. Ultimately, these findings also generate insights for interested parties when designing and evaluating complex digital health interventions.

## Appendix

Multimedia Appendix 1 Interview schedule/questions guide.

Multimedia Appendix 2 Themes and quotes.

## Abbreviations

BED: binge eating disorder
BN: bulimia nervosa
COREQ: Consolidated Criteria for Reporting Qualitative Research
ED: eating disorder
ISRCTN: International Standard Randomised Controlled Trial Number
mHealth: mobile health
NHS: National Health Service
NIHR: National Institute for Health Research
RCT: randomized controlled trial
TA: thematic analysis
UX: user experiences
