# Supplementary material for: Exploring Participants’ Experiences of a Web-Based Program for Bulimia and Binge Eating Disorder: Qualitative Study
Source: J Med Internet Res. 2020 Sep 23;22(9):e17880. doi: 10.2196/17880 (PMC7542406; doi:10.2196/17880)
Supplement: Multimedia Appendix 1 [file jmir_v22i9e17880_app1.docx]

**Multimedia Appendix 1. Interview Questions Guide**

**General Questions**

1. How are you now in general?
2. What experience do you have with regard to Internet (guided) self-help interventions (for any mental health conditions) apart from everyBody Plus?

**Acceptability**

1. What were your first thoughts when you heard about everyBody Plus?

**Expectations**

1. If you cast your mind back to before you started the programme: what were your hopes and expectations before starting the sessions?

*Possible variations:*

- How did you think this might work for you?
- Anything you looked forward to/ not looked forward to?

1. What has/ has not met your expectations?
2. How has this been different from any previous interventions you may have accessed for your eating disorder (face to face/ online/ book-based)?

**Content – General**

1. What changes (for the better/the worse), if any, have you noticed in yourself through taking part in the programme?

- eg. ED behaviours, outlook, feelings about future, relationship with others, routine etc

1. How, if any at all, have your view of the programme changed throughout the course of the sessions?
2. How has the programme helped/ not helped with you preparing for face-to-face therapy/ seeking treatment for your eating disorder?
3. What programme content has been the most helpful/ relevant to you?

- Is there anything that should be changed?
- Is there anything we missed out?

**Content – specific**

1. Which of the following has been most helpful & least helpful for you?

- Group messages
- Symptom diary
- Self-reflection diary
- Therapist support

1. How’s the experience of _______ (refer to above question) been?

- How can that be improved?

**Engagement [Format and user experience]**

1. What has been easy/ difficult for you in order to integrate the programme into your daily routine?

- Technology factors: eg. Notifications, user-interface, user experience
- Personal factors

1. Is there any way the programme can be improved to increase regular use?

- Eg. How it looks, how you interact with, layout, media use etc.

**Ending**

Anything else you would like the research team to know?
