# Supplementary material for: Exploring Participants’ Experiences of a Web-Based Program for Bulimia and Binge Eating Disorder: Qualitative Study
Source: J Med Internet Res. 2020 Sep 23;22(9):e17880. doi: 10.2196/17880 (PMC7542406; doi:10.2196/17880)
Supplement: Multimedia Appendix 2 [file jmir_v22i9e17880_app2.docx]

**Supplementary Material 1**

Table 1. Themes and quotes (GF= group forum, I= interview, P= participant, SF= in-Session feedback; themes are bolded)

| **Tailoring user journey** | I am finding the programme very stressful and, by not being able to cope with the amount of information, like I'm failing at it. (SF)  My perfectionist tendencies stressed with absorbing everything…I felt really frustrated some of the time because I couldn’t go at my own pace and there were such deep valuable concepts contained within the session that it was overwhelming to have to consider all of them within the timeframe. It was too much for me...(SF)  I also found the user experience really bad. It's like eating a lovely biscuit that has sand in it. It doesn't matter how nice the biscuit is, the sand ruins it. – (GF)  The first section on how to use the site is helpful, although I'm still puzzled about where the forum is. I'm hoping that when I go back to the home page it will be obvious. Or is the forum in the contact sections, and topics will be sent out for people to comment on? So that I am confused about. And also where the self-reflection diary is. Maybe I need to reread the how to use the site guide again! (SF)  I have a short attention span, so if I am tired, it sort of, I am okay, it's the last 10-15 minutes that sort of get me (P9, I)  No, it was really simple, yeah there's nothing, very simply and easy to understand. It wasn't like overwhelming to look at, just an easy and simple (P10, I) |
| --- | --- |
| Customisation | I find them (the notifications) quite useful, actually I have been quite busy in the week, maybe I have forgotten to fill out the symptom checker and I find that quite useful (P1, I)  I like that I am reminded that every week. I think that keeps it interactive because there's always different forum, with the discussion posts, so urm, and also the fact that you are kind of required to fill it in on a weekly basis so you kind of keep returning for it. (P6, I)  An ED affects every waking hour of the day - if online programmes are to be used they should fit round the user more, rather than the user having to fit round the programme [the programme currently did not allow them to customise the time of the notifications]. (SF)  Probably could text people, more modern way of getting through to people nowadays, seeing texts isn't it, that would be a way, maybe, if people chose to email contact but then could be ticked off from the onset. Sometimes not everyone got access to email, I don't often have chance to look at them as much as I would like to, but most people will have a phone. (P3, I)  I found that fine, I mean was able to, at one stage, in terms of the discussion notifications and things like that, I found them quite frequent, and I changed them to have them, kind of less frequently. I like that fact that I was able to make my own choices about how often to get to notifications and things like that (P8, I) |
| Personalisation | I tend to just do the reading and read through it instead of the audio. (P1, I)  I often ended up using the transcript, that were associated with them. That's just because sometimes I tend to take in things better by reading them instead of hearing them, and that but it was nice to have a range of different voices, and different kind of people, age in it, and feeding back. (P8, I)  they definitely helped, because I can't read text, it makes me lose my attention quicker. So it definitely put me on the phone and let it read it to me (P9, I)  I don't like about the user-friendliness is that if I close the page partway through, even if I do an intermediate save, next time I open the session in a new window I must start from the beginning and click through all the pages to where I previously was. It would be nice to have an easier way to go back to my previous position, or to go to particular pages more quickly, e.g. a contents page at the start of the session, with each page becoming available as you complete the session. (SF)  It would be helpful if the other sections - nutrition, body image, what are eating disorders - had shortcut buttons, so I could click to go the beginning of the section rather than having to scroll through the entire session to get to the pages I wanted to reread. (SF)  I also got annoyed at having to click through a lot of pages again. When you leave for a while it takes you back to the beginning. I do this on my phone so I can't click enter to move forward. I understand that you can't change this at the moment but any criticism would be incomplete without highlighting the usability problem. (SF)  Laptop, I couldn't get it to work on my phone.(P2, I)  I don't think you could access it on your mobile in the same way, it was just stripped. You have to go onto the computer to do some of it. Just trying to think of which. I remember, you could put some of it on your phone but you can't use it all on your phone, that can be a bit of a pain sometimes because you kind of think it would be a bit more private sometimes if you could just do it on your phone. But you couldn't, I had to log on to the computer in downstairs, the other room with family, made it slightly more difficult for accessing. (P7, I)  Personally no, I would keep using the laptop. Because, partly because you know, if you want to use the, do the mindfulness diary, for me, I am just not gonna to do that on my mobile, I know some people can type with that but personally I can't, so laptop works very well for me (P2, I)  And also, I didn't want to do it on the go. It's when I had a bit of time and space to think about it, if you see what I mean. Like I would do twitter when I am on the bus, but I wouldn't do this on the bus. I need to do it at home and before I go, and things like I do it on a Monday and that's it, and that's done. But I just begin to see that now, because it takes time to do the, processing. (P4, I)  I am always doing something on there, whereas actually going onto a laptop and doing some kind of regular, some sort of like a class or something, actually made it, gave it a bit more weight, made it feel like I was taking it a bit more seriously, so I personally found doing it via a laptop would probably be better in terms of my level of engagement (P8, I)  you do have an app, because when I am out, I don't take my laptop with me, my phone, I will just bring my phone, I won’t bring my laptop, so it does help in that sense, that I can just log in and do the main thing, like the diary, symptom tracker, get hold of people, so it does definitely help having an app. (P9, I) |
| **A flexible and “everyday” programme** | I moved around every year, I moved house, not necessarily moved far, but obviously when you are in a different area, you are obviously under the care of different like (district), so then I wasn't kind of like, able to sort of get a regular therapist or anything…Life got in the way. (P6, I)  If I could maybe download the exercises from the start of the paper, and have them in front of me, I might have, might have been encouraged to carry on with them. Because they weren't there in front of me in black and white, I didn't. (P4, I)  I found joining the programme because it touched on things in other aspects of your life, it made every day into that, part of it, whereas I found sometimes during therapy although, it became like a silo, separate from your normal life. Whereas this, because it became part of your normal life, you could think about it, think about reflecting on aspects of your life, whilst you are living it almost. (P7, I)  To have an option of using the support as in when needed as opposed to a fixed time each week. I really would struggle to go to a support with a particular time, so I like the flexibility of it. (P3, I) |
| **Reflections about their ED recovery** | The most useful thing was writing about my own experience. I know it well, after all these years of therapy, but it was cathartic to get it down in writing. (SF) |
| Cognitive/ emotional | I learnt about different aspects of their eating disorders (eating, body image, exercise, emotions) (SF)  Exercise - Even though I don’t over-exercise . I found it useful to learn about resting intervals (SF)  I can see that I have changed my attitude to exercise. It used to be about losing weight (even if I would then counteract that with my eating habits). Now it is about contributing to more positive mental and physical well-being. I think this is something I can carry with me going forwards. (P6, I)  Media - it made me think about my feelings towards myself and how the media films etc have affected me and why being judged as a teenager has contributed towards my eating disorder and being bullied as a teenager has made me the person I am today (SF)  I really liked this session, especially the letter writing part as it gave me the opportunity to really work through my emotions which is something I struggle to do and to be kind and forgiving of myself. (SF)  yeh and it showed me just how hard I am on myself and I didn’t realise, you know I didn’t realise that I was, you know I knew that I didn’t have very much self-esteem, but to see it on there and relate to a lot of what’s on there and like I said you get a lightbulb moment. (P12, I) |
| Interpersonal | I think I get less offended by what people say around me. I used to be negative about any comments about my weight or what I was eating, but I have learnt to not take that personally or at least tell them what they are saying is quite offensive, instead of going off binging I have actually dealt with it instead.  (P1, I)  I found the advice on actionable steps to improve interpersonal relationships very grounding and encouraging. (SF)  It was difficult going out for family meal for (my partner’s) mum and dad for Father's day. And I actually wore something, sort of, I managed to wear a dress, I actually dressed up, and just going out for dinner, quite emotional for me, cause I have never done it, I have never been able to do it, for a long time, for a long long time. (P9, I) |
| Behavioural | I think it's more practical, in the past when I had therapy about it, it's just been talking about things that this is actually talking about things but this has actually just given me exercises to do…I found that quite helpful, because you are putting it into practice. You are not just reading, you are doing something. (P1, I)  When I feel upset stressed, angry this week I will try the meditation techniques and see if that clears my mind. (SF)  I plan to set weekly goals for myself to be monitored the following weekend but to keep them small and achievable. Such as to eat 3 meals a day along with healthy snacks etc (SF)  I will try and use the diversion (distraction) such as ringing a friend, reading a book, just change and think of new ways to bring the cycle really. That's kind of what I have taken from it.(P3, I)  Mindful eating/ diet – I like this session because I liked the emphasis on the listening to your body sections, like hunger pangs and feeling full and stuff which ive been trying to do for a while now. (SF)  So I did, as a result of one of the exercises, I did finally get about, putting a mirror up, and I do kind of just thinking about taking a little bit pride in myself and feeling like I need to have, I wanna go out, feeling more comfortable, feeling a bit more confident in terms of as I am leaving the house, what helped me then, during the day, that sort of confidence. I think, I have tried to become a bit more confident just in terms of what I feel comfortable wearing, and not kind of needing to apologise for that, or finding things, finding clothing that makes me feel more confident. (P8, I)  Really made me feel motivated and got me thinking about the viscous circle of self-conscious avoidance behaviours and baby steps are needed (SF)  I mean personally, it has been quite positive, I have had some, probably the longest time of not binge eating since I have started bingeing. I think I ended up having like 6 weeks which I haven't binged at all which was like a massive achievement for me. (P3, I) |
| **Not a “one-size-fits-all”** | Personally, I think for this kind of programme, it should be made accessible to anybody who wants to do it, I really do think it’s a good programme. (P. F) |
| Perceived usefulness and relevance of the programme content | Perhaps, mirror exercise would be helpful for a group of people but not everyone, also mentioning that and also catering for people who, in terms of the themes of exercise, there could be people who overexercise, or don't exercise...(P2, I)  Mirror exercise would not result in me suddenly having an aha moment where I realise any part of my body is ok...because I am obese (SF)  The exercises again were not relevant to me, as they were clearly geared to people at a normal or low body weight, not for those of us who are fat. I can’t stand in front of a mirror and tell myself I am alright, when clearly I am not. (SF)  I can't look in a mirror at my body, hm, I felt to the girl who were very large, they must have found it more difficult, and the ones on the forum said they had done it, I think they were very brave doing it, but I couldn't do it. (SF)  The most challenging part of the programme were the mirror exercises. I truly did not expect for them to be so hard. At the same time, I am glad I did them. I know I have to accept myself at a healthy weight. My eating disorder stems, in part, from wanting to be at a weight which I can only maintain through unhealthy habits. (SF)  I know a lot of people were kind of saying it they really didn't find that helpful, you know, it made us feel quite bad about themselves, but for me, I didn't feel that way. That encouraged me to think about I am quite a negative personally about my appearance. I think I did quite helpful, it did me think I am not quite as bad a person I think I am really, and that other people could see me differently the way I see myself. (SF)  The most challenging aspect of the programme for me were the mirror exercises; at the beginning I did not even have a proper mirror up in my flat because I disliked looking at myself. While I still do not like this, I do think it has enabled me to start to challenge myself to identify aspects about my look that I like. It's also helped me identify tricks to make me feel more confident: knowing that I feel more confident leaving the house with a pair of shoes I like or with my hair styled how I like, helps me plan better for those days when I need that confidence. (SF)  I have finished this week's course pretty furious. This was twee and facile. Writing letters from bits of our body - really? Especially if the letters are meant to follow the example and have our thighs say how sad they are we don't like them. Come on. And for the rest: a lot of it seems based on the idea that we should just accept our body. Well, I'm currently walking past posters from Cancer Research telling me that OBESITY is one of the largest causes of cancer, and I'm meant to ignore that in favour of feeling good about my body, am I? (SF)  I feel the course has been totally dismissive of any problems that come with obesity. It is not represented in any of the examples given, any questions asked or any exercises offered. Obesity is not even mentioned in the list of possible complications and secondary damages of eating disorders (session 2) The authors might had it in mind while working on it but unfortunately, it doesn't come across as such.  (SF)  But the fact that I have got BED and (inaudible) the fact that I’m so heavy and so large and classed as morbidly obese I think there’s not enough on the course for people in my situation. (P12, I)  The other thing I want to say was, it seems slightly more geared towards younger women than older women, say those little videos and everything. That needs a little bit more, more younger…Getting old with an eating disorder is a different challenge in itself…I think I hear things about women my age with an eating disorder, it always feels a little bit like it's been written off, like there's no hope after a certain age if you have it for a certain amount of time that it will be with you for life, there's no kind of recovery as such. I mean that's my perception anyway. (P7, I)  There were no elderly women, there wasn’t, you know it was like if we’re going to have someone black lets have them light skinned black. You know, and I noticed that straight away and I thought is it just me and then when I read other comments it was very similar. It was like hang on a minute here, you’re telling us about self-image, mirror exercises, boosting self esteem and then look at what you’ve given us pictorially. (P11, I)  also the weird focus on attractiveness, which seemed to come up week after week. I probably need to go back and read the sessions to see if 'feeling attractive/unattractive in your body' did get mentioned as much as I felt it did. (SF)  Well I think otherwise, generally there are things which are beneficial, but I think a lot of the time on everyBody etc what we think about our body, I have a feeling that for people who purge etc, which I don't, so for me, i don't know how to explain it, but I feel that people who have problems with their bodies so that they overeat or whatever(P5, I)  I like that this session made me realise how my distorted body image is in many ways the byproduct of a distorted beauty ideal. Even though I am healthier, it is still frustrating how much these "perverse' standards still linger. Recognising it is part of a long socialisation process gives me a degree of comfort. (SF)  Very thorough and detailed and useful. The focus on external factors such as the media I found particularly interesting. (SF)  further most sufferers don't give a damn about the media portal of women and what an ideal body shape or weight is. (SF)  The problem of attractiveness, however, comes up again and again, session after session. (SF)  I think from the start adverting, oh, we can show a larger woman, and a slightly larger woman, and you know what, that's an idealistic, glamourised version of the problem, probably air-brushed as well. You know what, it's such a relief to just see ordinary pictures of women sometimes. I mean, if you go trying to say to somebody, you need to, you know, that actually are working towards acceptance of body, would you feel great to have your images, and I think that would be really helpful. You know, a picture of some real women for example, it's just so useful. (P2, I)  The example scenarios from Sarah and Aisha were again focused on bulimia and anorexia. There was nothing here for the fat binge eater. (SF)  I found them really engaging, I found them really useful to hear from their perspective, and to be able to learn more about their stories, going through the programme, I think it gave them kind of, yeah, I think that's a really helpful kind of touch (P8, I) |
| Different engagement styles in the group discussion forum | I am not a big girl, I am a normal size, I am trying to join in a conversation about how you feel about how you look when you know that there are people out there, that battling harder in a way, because they are so large. I just couldn't feel I could do it, so it's difficult when there's being such different from other people using the forum. (P4, I)  But I thought maybe the bit that as a group, I didn't think that many people got involved. There were few people talking, but not really everyone else. (P1, I)  But I think a lot of practical things, and you know, the sort of, that feeling of somebody being with you in the journey and cheering you on, I think that could be hugely valuable…I have to say I did also sometimes, if I also was having a bit of down time, I logged on to some of the forums to see what other people were up to in the middle of the night. (P2, I)  I do not feel so alone right now. I have struggled to explain things to my mental health co-ordinator regarding my symptoms. He just said 'oh it’s just comfort eating' and 'you are not serious enough for me to refer' to which really upset me, so in view of that this programme is helping me so far understand my behaviour. (SF)  It's quite a nice sort of feel not like alone and everything, the sort of thing that I don't really talk to anyone about, or that I have ever told anyone about, that normally I just have like, have to go everything solo, but now it's quite nice to know that there's a whole like other community of people who have been through the same experiences with me, but if I have a bad day, and I might log on, and just remind myself with things. (P6, I)  I think today I read one and it said that they’d not been on for a while because they’d been eating or whatever and someone had written take it one day at a time. And then I just wrote underneath you know take care of yourself, self-care, be kind. You know I try and write, really what I would have liked to hear when I was younger. (P11, I)  I didn't feel part of it (the online forum), I felt I was outside of the group, didn't feel I could take part…I know everybody on it sort of got a problem with binge eating, but everybody was different size, different circumstances, I just felt I didn't fit in anywhere. I didn't feel I could join in…When I was trying to join in and I couldn't, and not a very positive feedback. It put me off completely and so much so that I stopped even thinking of posting anything. (P4, I)  I was initially a bit nervous about getting involved in the online conversation, I was much more of a, a lurker, reading other people's comments and not actively engaging, the things I engaged myself was the programme and the activities, and I felt more confident to engaging in the discussion and kind of feeling like it's really useful to know how other people are doing and being able to have that level of connection? (P8, I)  I am a very anxious person. So I have stayed cleared on it, I sort of sit it on and was like, no. (P9, I)  I knew that wasn’t for me, although I did observe and read people thoughts and feelings and everyone's supporting each other, I know that I never wanted to participate in anything by writing anything or asking for support. I just quite like reading what's going on with other people. (P10, I) |
